# The complete chloroplast genome sequence of Magic Lily (*Lycoris squamigera*)

**DOI:** 10.1080/23802359.2018.1527193

**Published:** 2018-10-26

**Authors:** Seung Woo Jin, Jee Young Park, Shin-Jae Kang, Hyun-Seung Park, Hyeonah Shim, Taek Joo Lee, Jung Hwa Kang, Sang Hyun Sung, Tae-Jin Yang

**Affiliations:** a Department of Plant Science, Plant Genomics and Breeding Institute, Research Institute of Agriculture and Life Sciences, College of Agriculture and Life Sciences, Seoul National University, Seoul, Korea;; b Hantaek Botanical Garden, Yongin, Korea;; c College of Pharmacy and Research Institute of Pharmaceutical Science, Seoul National University, Seoul, Korea

**Keywords:** Chloroplast, genome sequence, *Lycoris squamigera*

## Abstract

Magic Lily (*Lycoris squamigera*), belonging to the Amaryllidaceae family, is cultivated for ornamental and medicinal purposes. To characterize its genomic information, we obtained the complete chloroplast genome sequence of *L. squamigera* by assembling Illumina whole genome sequence data. The complete chloroplast genome is 158,482 bp in length which is composed of four unique regions, a large single copy region (LSC) of 86,454 bp, a small single copy region (SSC) of 18,500 bp, and a pair of inverted repeats (IR) of 26,764 bp. The genome annotation predicted 159 genes including 105 protein-coding genes, 46 *tRNA* genes, and 8 *rRNA* genes. Phylogenetic tree analysis revealed that *L. squamigera* clustered with *Allium* species belonging to the Amaryllidaceae family.

Magic Lily (*Lycoris squamigera*) belongs to the Amaryllidaceae family, which contains more than 750 species (Friesen et al. 2006). It has been cultivated not only for its ornamental value but also for its pharmacologically active alkaloids such as galanthine and squamigine (Martin 1987, Jin et al. 2011). Although there are many studies focusing on their ingredients, there are few studies conducted on their genome including three complete chloroplast genomes in the Amaryllidaceae family (NC_035971.1, NC_031829.1; Filyushin et al. 2016 and KX683282; Kim et al. 2016). Due to sharing of similar phenotypes between *Lycoris* genus, classification among this genus is difficult (Yoo et al. 2011). Since chloroplast genomes are widely used for understanding genetic diversity and evolution of plants (Nguyen et al. 2018; Joh et al. 2017), understanding the chloroplast genome has great significance. In this study, we report the complete chloroplast genome sequence of *L. squamigera* by assembling whole-genome Illumina sequence data to understand its genome and provide concrete information on the phylogenetic relationship of this family.

The leaves of *L. squamigera* were collected from Hantaek Botanical Garden (Yongin, Republic of Korea, 37°05'41.6″N 127°24'23.4″E) for preparation of whole genome DNA. Sequencing was conducted by the Illumina Miseq Sequencing platform (Illumina, CA), and 1.3 Gb of sequence data was generated. Quality trimming and assembly of the reads were conducted by the *de novo* assembly of low coverage whole genome sequence (dnaLCW method) (Kim et al. 2015a,b) using a CLC genome assembler version 4.21 (CLC Inc., Aarhus, Denmark). Assembled contigs were joined into a single draft sequence by guidance of chloroplast genome of *Allium cepa* (KF28079) as a reference. Confirmation and correction of the draft sequence were manually done with mapping of raw reads and BLAST searches. The plastid genome was annotated with GeSeq (Tillich et al. 2017) and manually curated.

The total chloroplast genome size of *L. squamigera* was 158,482 bp (GenBank accession no. MH118290) which is composed of four typical chloroplast regions: a large single copy region (LSC) of 18,500 bp, a small single copy region (SSC) of 26,764 bp, and a pair of inverted repeat region of 26,764 bp. The plastid genome contains 159 genes including 105 protein-coding genes, 46 *tRNA* genes, and 8 *rRNA* genes. All of the genes are single copy genes excluding 25 protein-coding genes duplicated in the IR regions.

Phylogenetic analysis was performed with coding sequences of *L. squamigera* including eight chloroplast genomes previously reported in Asparagales using a neighbour-joining analysis of MEGA 7 (Kumar et al. 2016) with 1000 bootstrap replicates. All species were classified correctly into four groups: Amaryllidaceae, Orchidaceae, Asparagaceae, and Iridaceae (Figure 1). In this phylogenetic tree, *L. squamigera* was grouped together with six *Allium* species in the Amaryllidaceae family.

**Figure 1.**
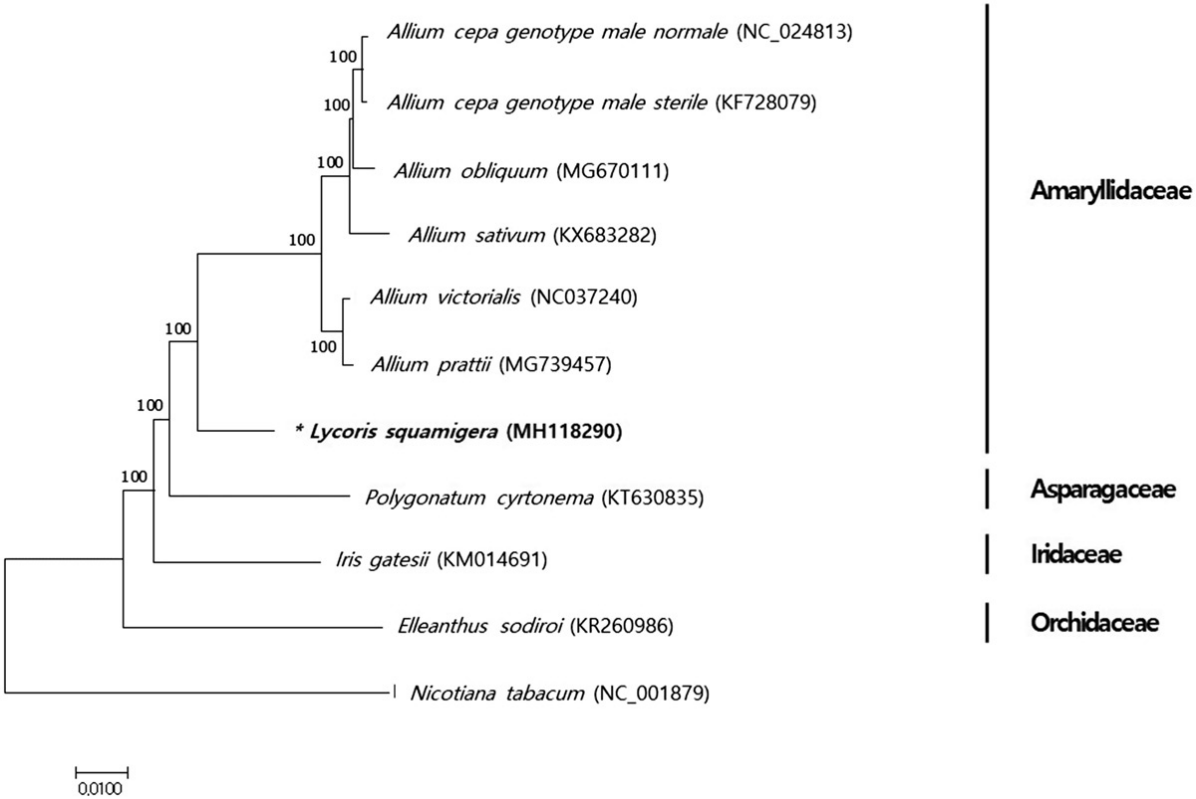
Phylogenetic relationship of *L. squamigera* with other 10 species of Asparagales. The tree was generated using 82 protein-coding gene sequences and full chloroplast genome sequences based on the neighbor-joining analysis of MEGA7. Numbers next to the tree indicate the bootstrap value from 1000 replicates. The sequence of *Nicotiana tabacum* was set as an outgroup.
